# Large‐scale distribution of bacterial communities in the Qaidam Basin of the Qinghai–Tibet Plateau

**DOI:** 10.1002/mbo3.909

**Published:** 2019-08-26

**Authors:** Rui Xing, Qing‐bo Gao, Fa‐qi Zhang, Jiu‐li Wang, Shi‐long Chen

**Affiliations:** ^1^ Key Laboratory of Adaptation and Evolution of Plateau Biota, Northwest Institute of Plateau Biology Chinese Academy of Sciences Xining Qinghai China; ^2^ Qinghai Provincial Key Laboratory of Crop Molecular Breeding Xining Qinghai China; ^3^ Qinghai Nationalities University Xining Qinghai China

**Keywords:** bacterial community, large scale, Qaidam Basin, Qinghai–Tibet Plateau

## Abstract

Many studies have investigated patterns of soil microbial communities over large spatial scales. However, these studies mainly focused on a few sites. Here, we studied the near‐surface (0–30 cm) soil microbial communities of 35 soil samples collected from most of the areas of the Qaidam Basin, which is the largest basin on the Qinghai–Tibet Plateau. A total of 32 phyla and 838 genera were detected from all the samples, in which Actinobacteria, Proteobacteria, Bacteroidetes, and Acidobacteria were the most dominant and cosmopolitan phyla. The most abundant phyla (relative abundance > 5%) detected in all 35 soil samples were also the most dominant, which could be explained by their great dispersal ability. The microbial community structures correlated strongly with variations in pH and Mg^2+^ and were distinct between the high Mg^2+^ content (>20 g/kg) samples and other samples (Acidobacteria, Actinobacteria, and Chloroflexi were significantly less abundant in the high Mg^2+^ content group, but the abundance of Firmicutes was significantly greater). Finally, the microbial spatial pattern was influenced by both the local environment and spatial distance, but environmental factors were the primary drivers of microbial spatial patterns in the Qaidam Basin.

## Graphical abstract

Our study of the large‐scale distribution of bacterial communities in the Qaidam Basin of Qinghai–Tibet plateau showed that the most dominant and cosmopolitan phyla in all the samples and both the local environment and spatial distance influence the microbial spatial pattern.

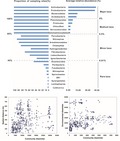

## 1 INTRODUCTION

The Qaidam Basin is a hyperarid intermontane basin that occupies a large area of the northeastern region of the Qinghai–Tibet Plateau (QTP), which covers a surface area of approximately 120,000 km^2^ and possesses the greatest reserves of lithium, magnesium, potassium, and sodium in China. This region is sensitive to global climate change, and the average annual temperature in the Qaidam Basin warmed at a rate of approximately 0.6°C/decade during 1982–2003 (Zeng & Yang, 2009). Annual precipitation in the Qaidam Basin is <50 mm/year, but the potential evaporation is approximately 3,000 mm/year, making the area extremely arid (Li et al., 2010). One‐third of the basin is covered by saline lakes and desert and is bordered by the Altyn Mountains, the Qilian Mountains and the Kunlun Range (Xia, Zhang, Yuan, Fan, & Zhang, 2001). The basin can be divided into three regions: the depression area to the east, the depression area to the west, and the broken block belts to the north. The altitude of this basin ranges between 2,600 and 3,000 m, which is comparatively lower than adjacent regions. The Qaidam Basin is the highest basin in China, and it forms a transitional region from the platform of the QTP (5,000 m) to the Qaidam Basin (3,000 m) and finally to the edge of the QTP (approximately 1,500 m) (Chen, Chen, & Nábelek, 1999).

Because of its richness in natural resources, studies of the microbial communities in the Qaidam Basin have mostly focused on salt lakes and gas fields. These studies focused exclusively on microbial communities and diversity in the salt lakes by isolating halophilic bacteria and studying microbial activity related to the formation of water and gas (Chen, Shuai, Osadetz, Hamblin, & Grasby, 2015; Duan et al., 2011; Han et al., 2017; Jiang, Xue, & Ma, 2015; Shen, 2017; Wang et al., 2014; Zhao et al., 2013). Soil microbes play an important role in maintaining soil quality and influencing nutrient availability. Bacteria compose a major part of the biodiversity in soils and play a major role in maintaining soil processes, which is crucial to maintaining the functioning of terrestrial ecosystems (Griffiths et al., 2011). Therefore, it is necessary to study the soil bacterial community and its driving factors.

The large‐scale soil bacterial community distribution has been well‐characterized by many studies. For example, surface soil bacteria can disperse globally (Green, Bohannan, & Whitaker, 2008), and bacterial community structure can be influenced by water and nutrient content (Hansel, Fendorf, Jardine, & Francis, 2009), soil pH (Griffiths et al., 2011) and soil temperature (Miller, Strong, Jones, & Ungerer, 2009), soil C/N ratios (Xiong et al., 2010), and moisture (Angel, Soares, Ungar, & Gillor, 2010). However, the abovementioned studies mainly focused only on a few sites (Fierer & Jackson, 2006; Griffiths et al., 2011; Lauber, Hamady, Knight, & Fierer, 2009). More recently, additional studies have found that spatial distance plays a vital role in influencing the distribution of microbial communities. These studies found that historical events (e.g., dispersal limitation, spatial distance, and past environmental conditions) and the current environment had significant impacts on large‐scale soil microbial distributions. For example, Fierer and Jackson (2006) found that, over large spatial scales, soil pH is the critical factor that shapes the microbial community (Fierer & Jackson, 2006). Alban and James (2007) found that spatial distance is likely correlated with microbial communities (Alban & James, 2007). However, a study of the pattern of soil bacterial communities in the Qaidam Basin on a large scale has been lacking until now.

Qaidam means “cornucopia” in Mongolian; this area, without any roads crossing it, may contain distinct soil bacterial communities compared to other areas in the QTP due to its extreme soil conditions. The objectives of the present study were (a) to use the Illumina HiSeq 2500 sequencing platform to analyze soil samples taken at a depth of 0–30 cm from 35 sites, which cover most regions of the basin and to compare the soil bacterial communities in this unique area; (b) to identify the driving factors that influence soil microbial community composition; and (c) to determine whether dispersal limitation or soil geochemistry operates more strongly on the composition of bacterial communities.

## 2 MATERIALS AND METHODS

**Figure A1:**
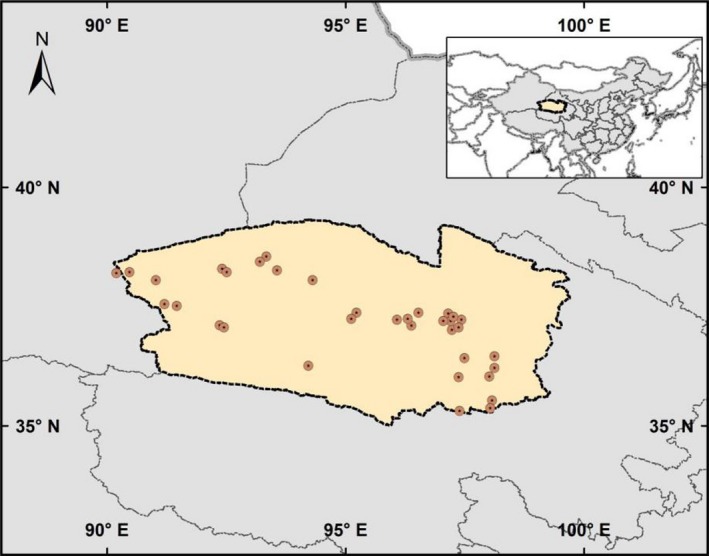
Thirty‐five sampling sites in the Qaidam Basin of Qinghai–Tibet plateau. The distance between sites is at least 10 km, and the altitude between sites is between 2,708 and 4,284 m

Soil samples were collected at 35 sites between June and July 2017, with the distance between sites ranging from 13 to 737 km (~100,000 km^2^), in the Qaidam Basin, Qinghai Province, China (Table A1 and Figure A1). At each site, we selected a single soil sample that was mixed from five sites located in a 100 m^2^ plot. For chemical analysis, all the samples were passed through a 2‐mm screen after being air‐dried. Total soil pH was determined using a pH monitor (Leici PHS‐3c). Electric conductivity (EC) and salt content (SC) were analyzed using a Leici DDSJ‐319L (1:5 water extraction). The contents of Mg^2+^, K^+^, and Na^+^ (in 1 M NH_4_‐acetate pH 7) were analyzed using an ICP‐AES (GDC Integra XMP). The contents of Cl^−^, ${\text{CO}}_{3}^{2-}$, and ${\text{HCO}}_{3}^{-}$ were analyzed using titration methods (Klute, 1986).

### 2.1 DNA extraction and HiSeq sequencing

Genomic DNA was extracted from 0.5 g of soil using a FastDNA SPIN Kit for soil (MP Biomedicals) and stored at −40°C. The DNA samples were then frozen‐transported to the laboratory of Sangon Biotech Co., Ltd., and analyzed using the Illumina HiSeq platform. The primers 515F (5′‐ GTG CCA GCM GCC GCG GTA A) and 806R (5′‐GGA CTA CHV GGG TWT CTA AT) were used to amplify the V4 hypervariable region of bacterial 16S *rRNA* (Caporaso et al., 2011). The thermal cycling conditions consisted of initial denaturation at 94°C for 180 s, followed by 30 cycles of denaturation at 94°C for 30 s, annealing at 50°C for 30 s, and elongation at 72°C for 60 s, and finally, the cycling was completed at 72°C for 7 min. The KAPA Library Preparation Kit (Kapa) was used to generate the sequencing library, and quantification was performed using an Agilent Bioanalyzer 2100 system (Agilent Technologies). Sequencing was performed on an Illumina HiSeqPE250 platform (Illumina). The sequence data associated with this study were submitted to GenBank under the accession number PRJNA513449.

### 2.2 Data analysis

The raw sequence data were analyzed by QIIME2 (version 2018.4) pipeline (Caporaso et al., 2010). Reads with quality scores below 20 or shorter than 230 bp were removed and then clustered into operational taxonomic units (OTUs) using UCLUST with a 97% similarity threshold based on the DADA2 algorithm (Callahan et al., 2016). The taxonomy of the OTUs was analyzed by the RDP Classifier against the Silva rRNA gene database (https://www.arb-silva.de/) with a confidence threshold of 80% (Wang, Garrity, Tiedje, & Cole, 2007). The software STAMP was used to compare the relative abundances of bacteria among the different groups based on analysis of variance (ANOVA) with a significance level of *p* < .05, and Tukey–Kramer's post hoc test (Parks, Tyson, Hugenholtz, & Beiko, 2014) was used to investigate differences between groups. Beta‐diversity indices between samples and principal coordinate analysis (PCoA) were determined based on weighted and unweighted UniFrac distance matrices (Lozupone & Knight, 2005). The relationships between bacterial diversity and soil properties were compared using SPSS 20.0. The Mantel test results were calculated using the microbial dissimilarity matrix (Bray–Curtis method) and environmental dissimilarity matrix (Euclidean method). Additionally, only variables with no auto‐correlation were included in calculating the environmental dissimilarity. Nonmetric multidimensional scaling (NMDS) ordinations were used to visualize the bacterial community structure based on the Bray–Curtis dissimilarity matrices. The relationship between microbial community data and soil variables was determined by Canonical correspondence analysis (CCA). The Mantel test, NMDS, and CCA were all performed using the vegan package in R software (Simpson, Solymos, Stevens, & Wagner, 2015). To compare bacterial diversity between samples, a UniFrac tree was constructed using Fast UniFrac (Bamberger & Lowe, 2010).

## 3 RESULTS

**Figure 1:**
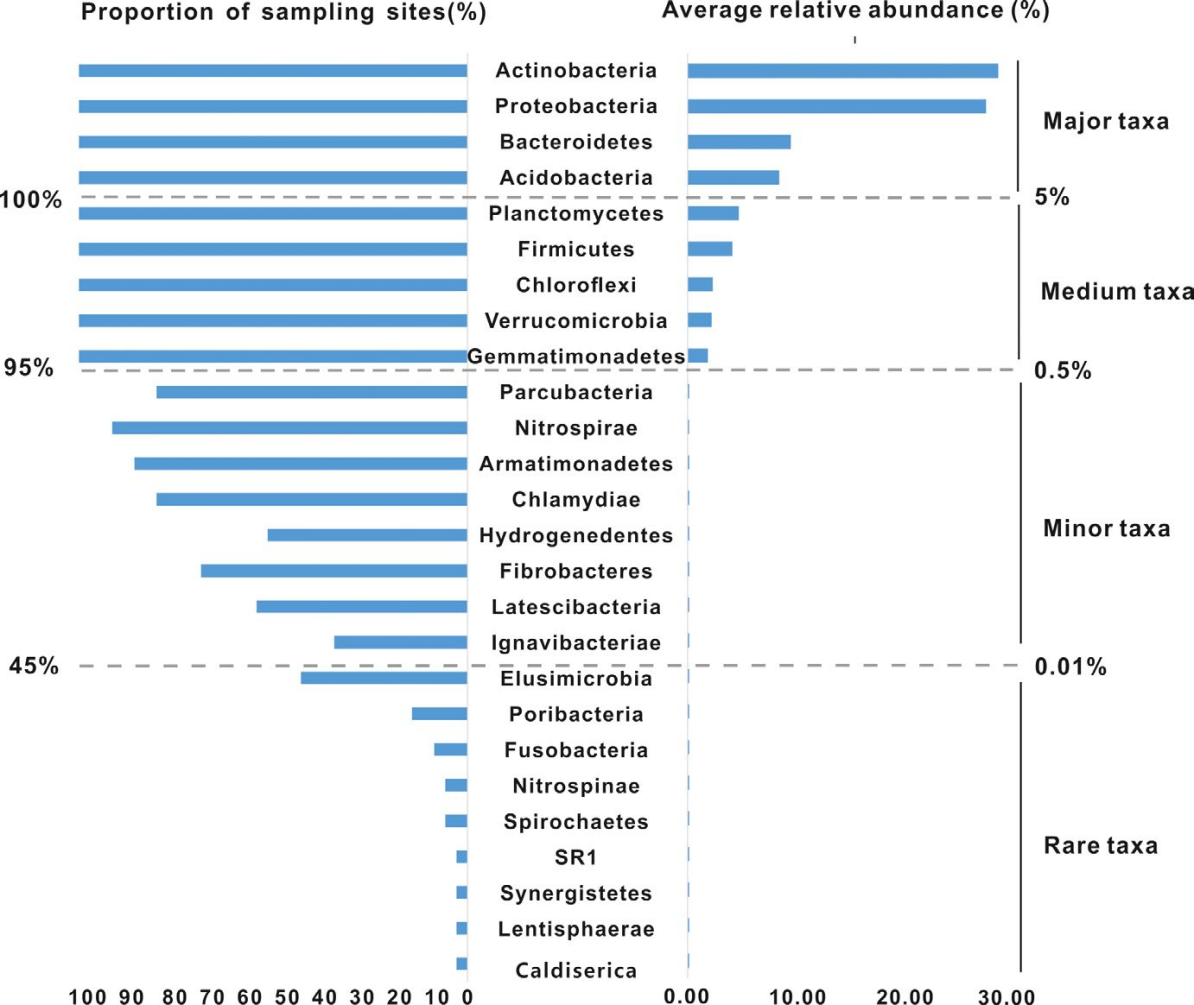
Representativeness of bacterial phyla in Qaidam Basin soils. Left: The proportion of sampling sites where phyla were present. Right: The average relative abundance of the phyla

**Figure 2:**
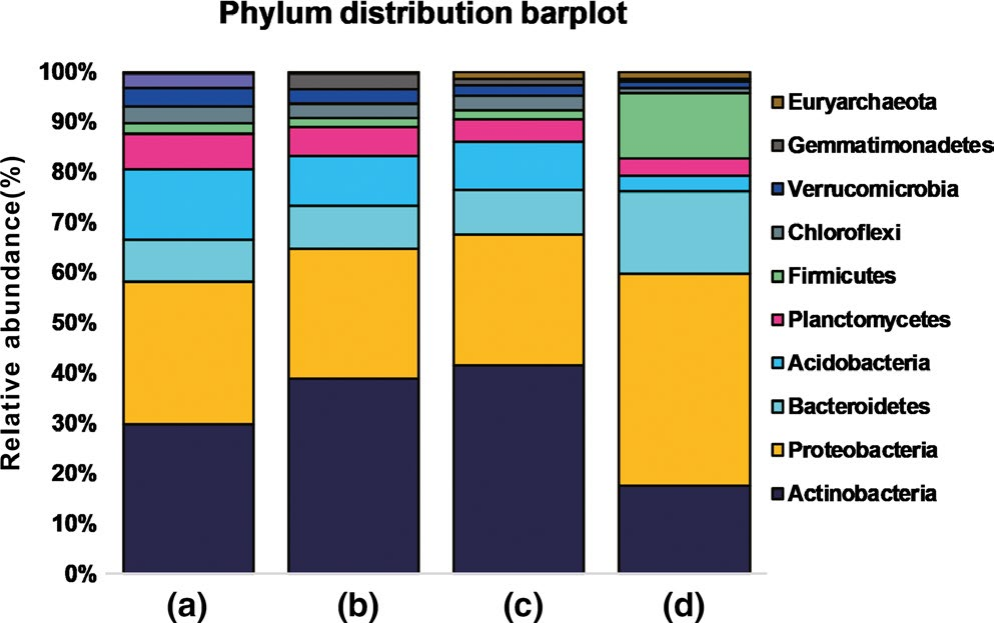
Relative abundance of the dominant bacterial phyla across the soils. Soils are grouped by Mg^2+^ content. (A: 0.77–5.24 g/kg, B: 6.22–11.74 g/kg, C: 12.14–18.72 g/kg, D: 20.15–36.15 g/kg)

A total of 2,186,792 16S rRNA V4 sequences were obtained from 35 soil samples (depth: 0–30 cm), and 144,038 OTUs were annotated (97% identity) from these data. Among the 32 phyla and 838 genera, 29 bacterial phyla and 667 genera were detected in at least two soil samples. The 32 phyla could be divided into four groups based on their average relative abundances and ubiquity. In the major and medium groups, eight of the phyla were found in all soil samples, with an average relative abundance ranging from 28.8% to 1.9% (Figure 1). Actinobacteria, Proteobacteria, Bacteroidetes, and Acidobacteria were the most dominant and cosmopolitan phyla (Figures 1 and 2). In contrast, the rare phyla (for instance, Elusimicrobia, Poribacteria, and Fusobacteria) were detected in fewer than half of the soil samples. There were 35 dominant genera found belonging to 8 major and medium phyla. The most abundant genus was *Sphingomonas*, with an average relative abundance of 5.02%. All 35 dominant genera occurred in 91.8% of the samples (Table S1).

**Figure 3:**
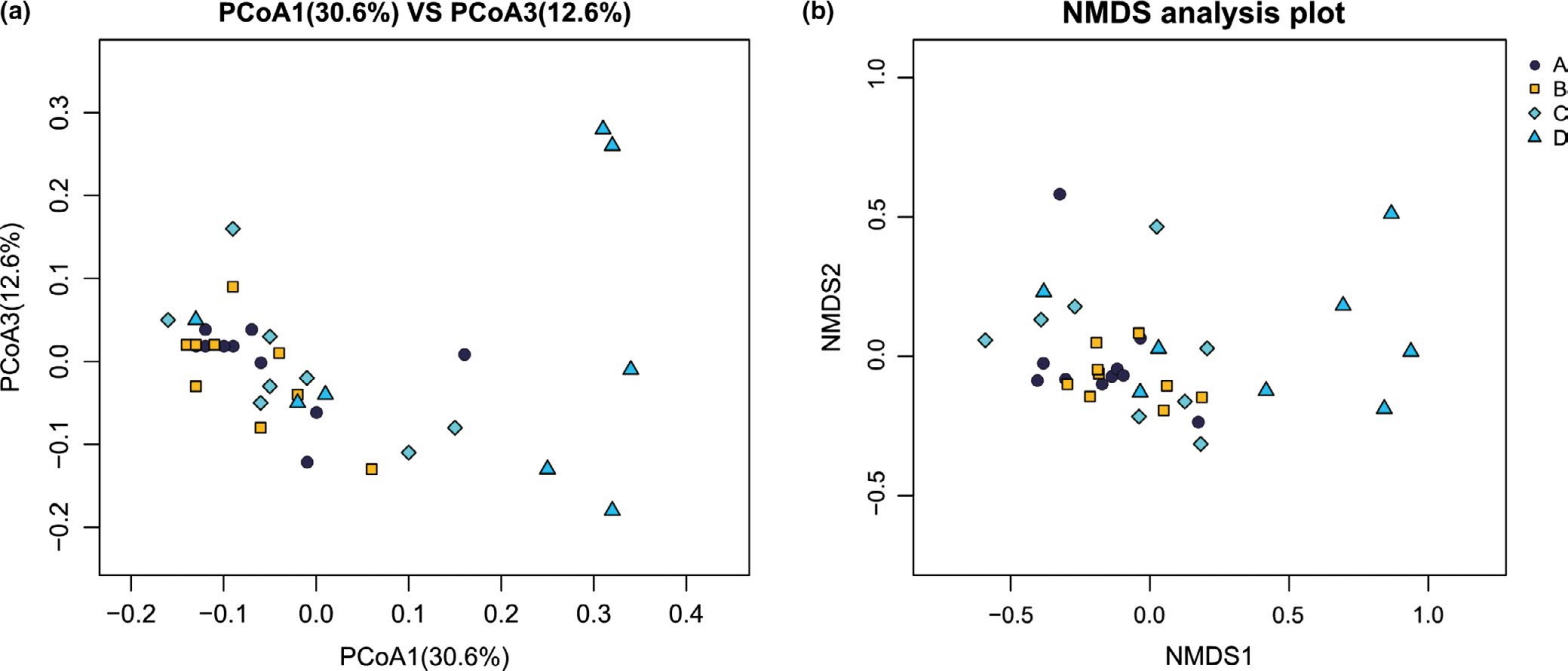
Principal coordinate analysis (PCoA) based on weighted (a) and nonmetric multidimensional scaling (NMDS) (b) showed that bacterial community changed along the Mg^2+^ gradient in Qaidam Basin. (A: 0.77–5.24 g/kg, B: 6.22–11.74 g/kg, C:12.14–18.72 g/kg, D: 20.15–36.15 g/kg)

**Figure A2:**
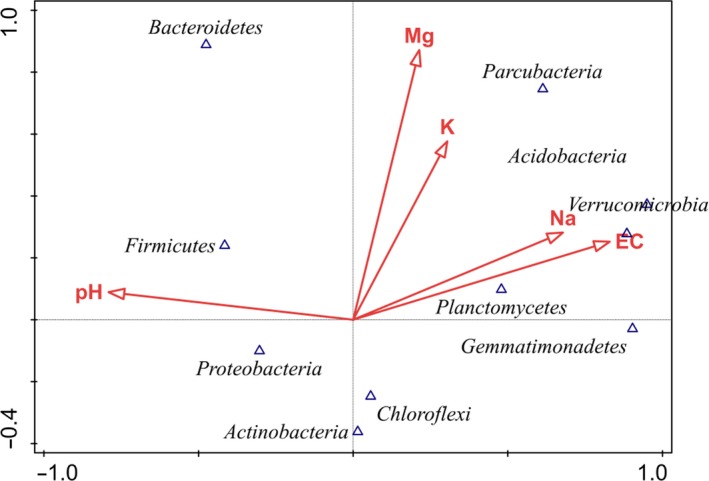
Canonical correspondence analysis (CCA) of 16S gene data and geochemical parameters

**Figure A3:**
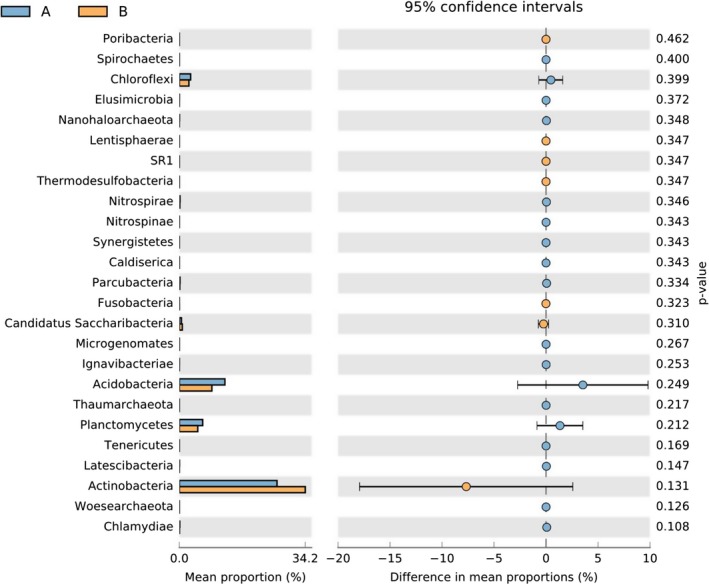
The bacterial phyla that were statistically significantly different (*p* < .05) between A and B

**Figure A4:**
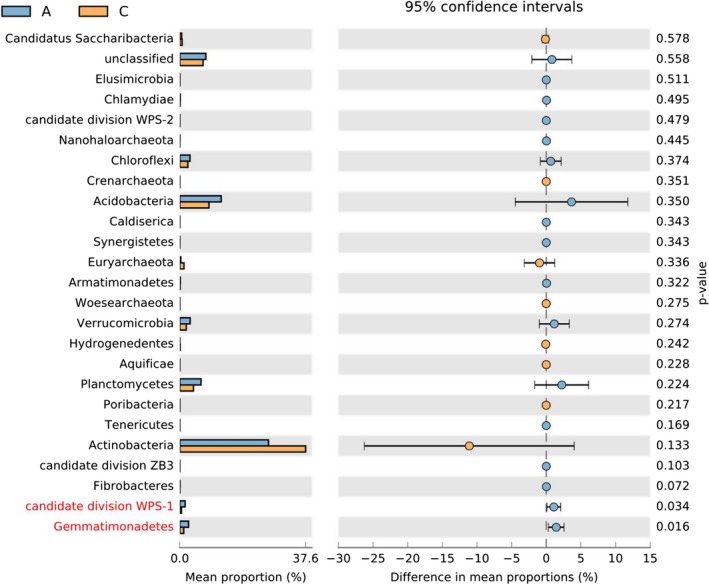
The bacterial phyla that were statistically significantly different (*p* < .05) between A and C

**Figure A5:**
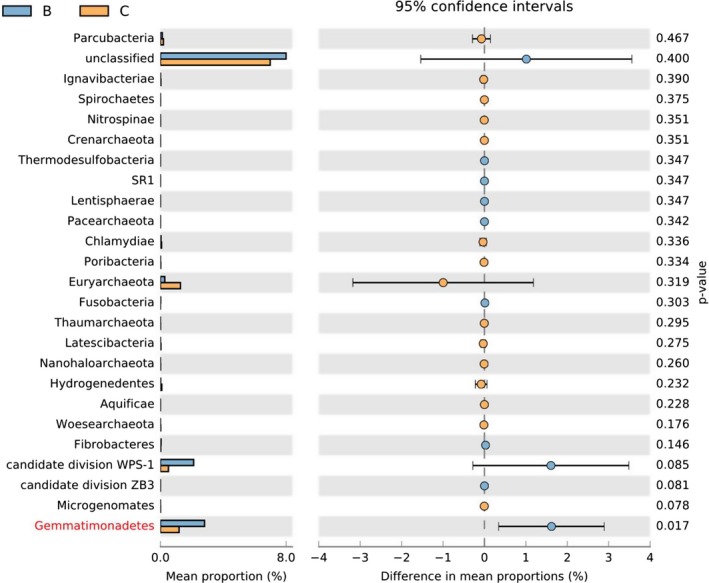
The bacterial phyla that were statistically significantly different (*p* < .05) between B and C

**Figure A6:**
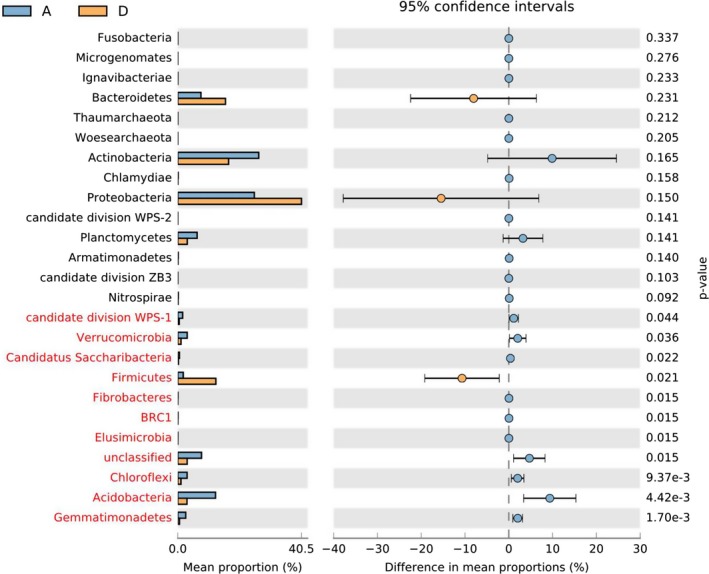
The bacterial phyla that were statistically significantly different (*p* < .05) between A and D

**Figure A7:**
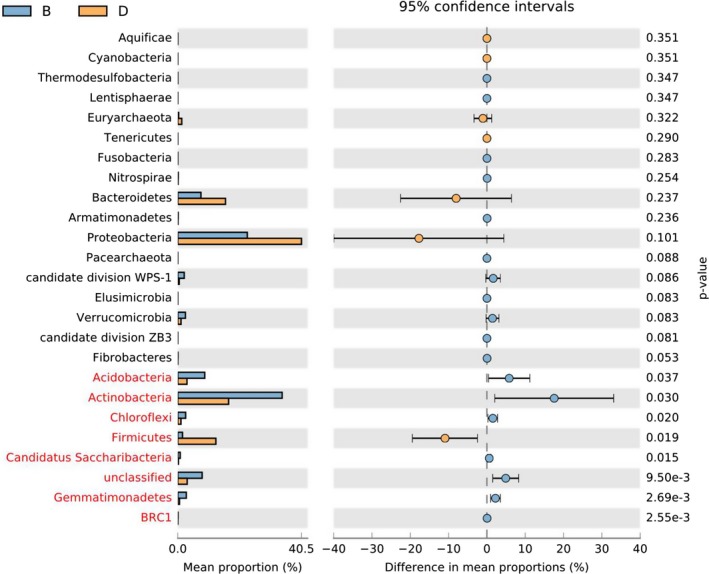
The bacterial phyla that were statistically significantly different (*p* < .05) between B and D

**Figure A8:**
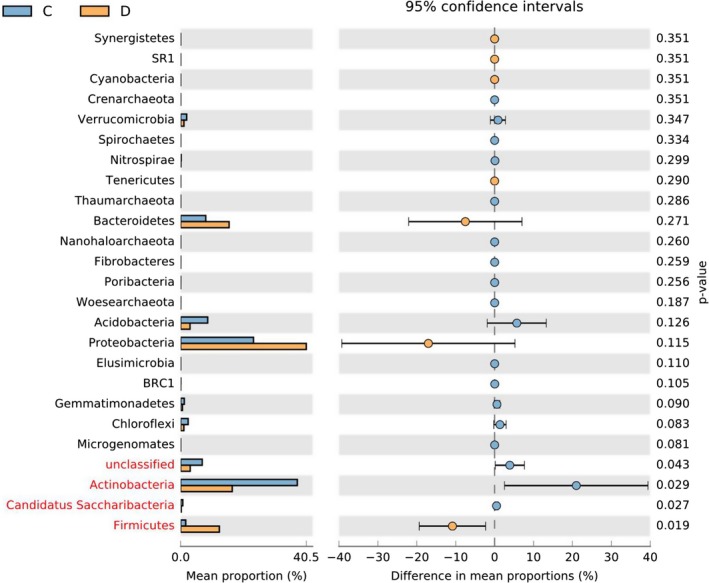
The bacterial phyla that were statistically significantly different (*p* < .05) between C and D

**Figure A9:**
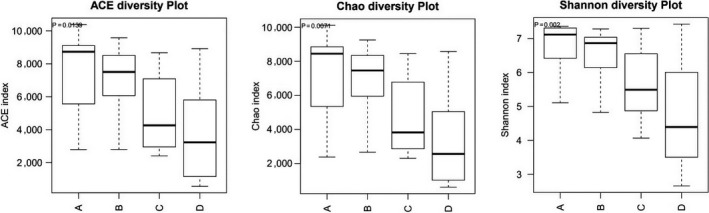
ACE, Chao1, and Shannon diversity indices

**Figure A10:**
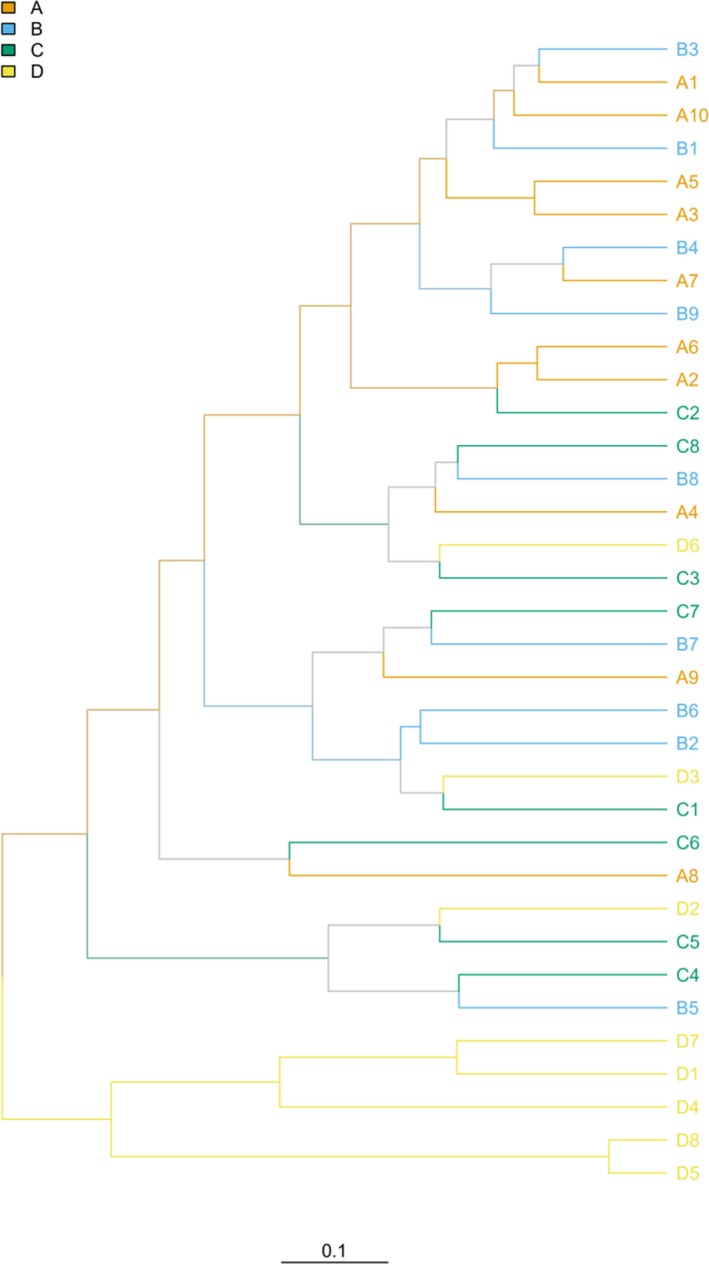
The unifrac tree of all the samples

Canonical correspondence analysis was used to identify the influence of soil properties on bacterial community variation among sites. The results showed that the bacterial community could best be explained by Mg^2+^ and pH levels (Figure A2). Based on the CCA, we investigated the change in the composition of bacterial communities along the Mg^2+^ gradient. The results showed that, as the soil Mg^2+^ content increased from 0.77 to 18.71 g/kg, only the abundance of Gemmatimonadetes differed significantly among groups (A, B, and C) (Figure 2, Figures A3, A4, A5). When the soil Mg^2+^ content increased beyond 20.15 g/kg, greater differences in microbial community composition were detected; for example, the abundances of Acidobacteria, Actinobacteria, and Chloroflexi were significantly lower in Group D, but the abundance of Firmicutes was significantly higher in that group (Figure 2, Figures A6, A7, A8). In addition, the ACE (*p* = .0139), Chao1 (*p* = .0071), and Shannon diversity (*p* = .0088) indices of Group D were significantly lower than in the other groups (Figure A9 and Table A2). Meanwhile, the NMDS and PCoA analysis confirmed that the bacterial community was distributed along the Mg^2+^ gradient (Figure 3), and the unifrac tree of all of the samples also showed that almost all of the soil samples of which the Mg^2+^ content was >20.15 g/kg (Group D) were separated from other samples (Figure A10).

**Figure 4:**
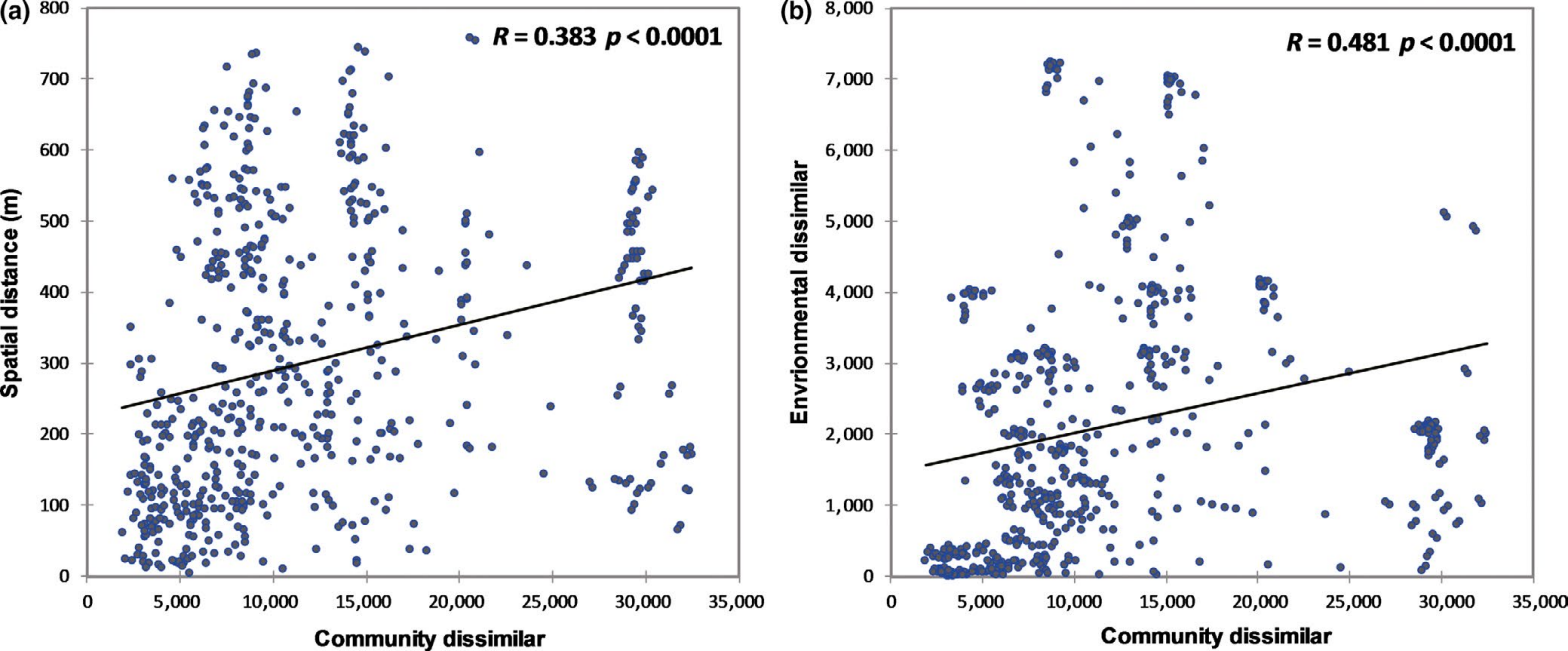
Relationships between bacterial community, spatial distance, and environmental distance

The Mantel tests were used to examine the influence of environmental factors and spatial distance of the microbial community over the large scale of the Qaidam Basin. Our results showed that distance factors (*p* < .001 *R* = .383) had a weaker correlation with the soil bacterial dissimilarities than measured soil variables (*p* < .001 *R* = .481) (Figure 4).

## 4 DISCUSSION

The soil is the most important habitat for bacteria in the terrestrial ecosystem; there are 10^9^–10^10^ bacterial cells in a single gram of soil (Griffiths et al., 2016). Many studies over the past decade have shown that some bacterial taxa are distributed in a restricted range of environmental conditions. Other taxa are cosmopolitan and can be found in more diverse environmental conditions. In the present study, the most abundant phyla (Actinobacteria, Proteobacteria, Bacteroidetes, Acidobacteria, Planctomycetes, Firmicutes, Chloroflexi, Verrucomicrobia, and Gemmatimonadetes) detected in all 35 soil samples from an area almost 100,000 km^2^ in the Qaidam Basin were also the most dominant. The dominant bacterial phyla (with a relative abundance > 5%) in the soil samples of the Qaidam Basin were also found to be dominant phyla in other soils (Constancias et al., 2015), oceans (Sunagawa et al., 2015), and in studies of mammalian gut microbiota (Donaldson, Lee, & Mazmanian, 2015), reinforcing the hypothesis that “Everything is everywhere, but the environment selects” proposed by Baas Becking (Bass Becking, 1934). Two possible reasons can explain this phenomenon: The first reason is that it is easier to detect abundant microorganisms with current techniques, and the other reason is that the bacteria with large population sizes have greater dispersal ability. The cosmopolitan phyla, such as Actinobacteria and Bacteroidetes, are dispersed by aerosolized soil dust (Barberán, Henley, Fierer, & Casamayor, 2014) and can colonize new environments. Firmicutes can survive in extreme environments due to its resistant physiological features. In contrast, we observed less cosmopolitan distributions of minor and rare phyla, which could be related to their limited abilities to migrate (Galand, Casamayor, Kirchman, & Lovejoy, 2009).

Microbial communities in the soil are strongly shaped by soil properties, such as available carbon, pH, and moisture (Chu et al., 2010; Fierer, Schimel, & Holden, 2003). In the current study, Mg^2+^ and pH levels in the soil of the Qaidam Basin had a strong influence in shaping the bacterial community structures. Many studies have shown that soil pH is one of the key factors influencing microbial community structure in soils (Fierer & Jackson, 2006). However, attention paid to the influence of Mg^2+^ was limited in such studies. Mg^2+^ is the most abundant divalent cation in living cells and the second most abundant cation. Mg^2+^ plays important roles in the cytoplasm and phospholipid head groups (Romani & Scarpa, 2000); thus, mechanisms to maintain physiological levels of Mg^2+^ are necessary. In our study, the abundance of Firmicutes was significantly greater in the high Mg^2+^ environment (>20 g/kg), because it can produce endospores and therefore can survive extreme conditions. Meanwhile, the abundance of Proteobacteria was also greater in high Mg^2+^ soil samples. This is a very interesting finding because members of this phylum contain a great number of halophilic bacteria, especially the Halomonadaceae family (Oren, 2002). This result is confirmed in previous studies where the most extreme and moderate halophiles are found in subgroups of Proteobacteria and Firmicutes, and similar bacterial community structures have been reported in several different high salinity environments: (a) The salinity of Dead Sea water is extremely high (34%), and the most abundant phyla found in the Dead Sea were Firmicutes and Proteobacteria (Jacob, Hussein, Mak, & Cornelison, 2017); (b) a study that investigated the microbial community structure in hypersaline soils (Mg^2+^ content >1,000 mg/kg) found that Proteobacteria was the dominant phylum (Hollister et al., 2010); and (c) another study in a cold and alkaline ecological niche (Mg^2+^ content was 28.7 g/m^3^ at a depth of 85 m) in the submarine waters of Greenland also found that Proteobacteria, Firmicutes, and Cyanobacteria were the dominant phyla present. In addition, some novel cold‐active enzymes have also been found (Stougaard, Jørgensen, Johnsen, & Hansen, 2010). Our results reveal that the Qaidam Basin contains a great number of halophilic and halotolerant bacteria that may be potential industrial resources; therefore, additional studies to evaluate such bacteria are needed.

To determine whether dispersal limitation or environment condition is the key factor that influences the distribution of soil bacteria in the Qaidam Basin, the relationships among the bacterial communities, spatial distances, and environments were compared. Most previous studies indicated that the patterns of spatial differences in microbial communities were influenced by both dispersal limitation and variation in historic environmental conditions (Chu et al., 2010; Jaechang & Tiedje, 2000; Martiny, Eisen, Penn, Allison, & Hornerdevine, 2011). However, other studies have shown that the environment plays a vital role in shaping the soil bacterial community (Fierer & Jackson, 2006; Griffiths et al., 2011). Our results show that both local environment and spatial distance influence microbial community structure, but the environment is the stronger driving factor affecting bacterial community structure in the Qaidam Basin. This result is similar to that of the study investigating the bacterial community in the western Tibetan Plateau (Chu et al., 2016).

Our study investigated the distribution of soil bacterial across the Qaidam Basin of the QTP. Actinobacteria, Proteobacteria, Bacteroidetes, and Acidobacteria were the most dominant and cosmopolitan phyla. The structure of the bacterial community could be best explained by pH and Mg^2+^ levels in the soil and was significantly different from other groups that exist in conditions of high Mg^2+^ content. Finally, the environment is the primary driver of bacterial community structure in the Qaidam Basin.

## CONFLICT OF INTERESTS

All authors declare no conflict of interests.

## AUTHOR CONTRIBUTIONS

Rui XING was the primary investigator of the original clinical study. Faqi ZHANG, Qingbo GAO and Jiuli WANG were involved in field work and laboratory analyses. Shilong CHEN was involved in supervision. All authors take responsibility for the reliability and accuracy of data, data analyses, and approval of the final version of the manuscript.

## ETHICS STATEMENT

None required.

## Data Availability

The raw sequencing read dataset was deposited at GenBank under the project accession number PRJNA513449.

## APPENDIX 1

**Table A1:** Sample site information and soil physicochemical properties

Site	Longitude	Latitude	pH	EC (μs/cm)	SC (%)	${\text{HCO}}_{3}^{-}$ (g/kg)	CI^−^ (mg/kg)	K^+^ (g/kg)	Na^+^ (g/kg)	Mg^2+^ (g/kg)
Q1	97.43	37.22	7.93	2,120	1.654	0.043	0.6	1.83	4.38	0.77
Q2	97.15	37.35	8.70	2,220	1.732	0.256	2.8	5.21	9.64	1.35
Q3	96.38	37.10	7.97	11,750	9.165	0.060	11.1	2.72	10.25	1.94
Q4	97.23	37.01	8.30	3,270	2.551	0.051	10.2	4.89	5.37	3.70
Q5	96.30	37.24	7.78	1,480	1.154	0.068	88.8	6.82	26.71	3.76
Q6	97.39	35.31	7.50	4,150	3.237	0.102	0.3	10.17	8.38	4.21
Q7	98.12	36.21	7.70	3,240	2.527	0.068	28.4	6.90	23.98	4.35
Q8	94.22	36.26	7.26	7,300	5.694	0.043	53.3	4.15	34.97	4.36
Q9	92.36	37.11	7.66	3,200	2.496	0.051	0	19.17	82.98	4.49
Q10	96.08	37.22	7.65	5,100	3.978	0.060	0	11.52	40.12	5.24
Q11	97.37	36.02	8.19	4,240	3.307	0.068	19.5	7.34	24.68	6.22
Q12	91.20	37.55	7.50	7,550	5.889	0.077	1.7	5.66	2.25	6.45
Q13	97.49	36.42	7.32	3,550	2.769	0.051	67.5	6.82	38.61	6.69
Q14	98.01	36.03	7.15	2,200	1.716	0.085	9.1	18.75	10.81	6.92
Q15	93.56	38.26	8.33	1,902	1.484	0.051	8.8	9.22	15.59	7.42
Q16	92.45	37.06	8.05	1,465	1.143	0.068	461.5	10.23	38.67	10.54
Q17	97.20	37.20	7.6	1,092	0.852	0.043	369.2	8.24	15.17	11.07
Q18	97.37	37.06	8.40	300	0.234	0.111	0	17.89	55.31	11.47
Q19	96.53	37.37	8.25	338	0.264	0.145	0	13.36	30.98	11.74
Q20	90.19	38.20	7.92	100.4	0.078	0.053	0.3	8.74	4.42	12.14
Q21	97.26	37.29	6.55	78.2	0.061	0.079	102	10.99	23.54	12.42
Q22	98.12	36.46	8.04	633	0.494	0.120	0	80.63	92.26	13.85
Q23	93.34	38.55	8.25	118.7	0.093	0.099	0	4.76	14.38	14.10
Q24	90.47	38.22	7.91	430	0.335	0.231	29.5	11.13	23.01	14.87
Q25	95.23	37.37	8.30	154	0.120	0.094	816.5	22.84	79.20	18.02
Q26	91.02	38.05	7.61	2,770	2.161	0.043	816.5	14.62	67.75	18.35
Q27	98.07	35.53	8.30	59.8	0.047	0.043	1,349	30.84	82.20	18.72
Q28	94.31	38.05	8.41	156	0.122	0.034	372.8	6.50	66.64	20.15
Q29	95.12	37.24	7.40	2,130	1.661	0.068	0	36.91	10.68	20.85
Q30	97.05	37.20	7.80	4,100	3.198	0.099	423	26.03	54.51	22.94
Q31	93.20	38.44	8.31	146	0.114	0.120	216.6	9.87	42.13	24.04
Q32	92.41	38.29	8.15	127	0.099	0.094	1,043	14.01	43.27	26.94
Q33	98.03	35.37	8.12	136.4	0.106	0.102	149.1	18.32	55.02	27.20
Q34	91.46	37.51	7.77	494	0.385	0.119	0	8.89	36.85	34.01
Q35	92.51	38.22	7.48	1,257	0.980	0.051	0	22.94	50.68	36.15

Abbreviations: EC, Electric conductivity; SC, salt content.

**Table A2:** Reads, OTU numbers, and alpha diversity index of 35 samples collected from the Qaidam Basin

Site	Reads number	OTU number	Shannon index	ACE index	Chao1 index	Simpson
Q1	66,504	6,558	7.13	9,227.39	8,853.52	0.0035
Q2	60,324	6,201	7.10	8,945.84	8,777.89	0.0035
Q3	51,813	6,310	7.35	8,779.35	8,457.39	0.0027
Q4	55,967	6,162	7.27	8,682.41	8,439.99	0.0034
Q5	62,115	4,028	6.42	5,347.32	5,157.79	0.0082
Q6	69,864	5,539	6.87	8,039.34	7,989.34	0.0044
Q7	59,267	6,880	7.36	10,385.09	10,114.42	0.0022
Q8	51,803	6,295	7.31	9,108.39	8,875.73	0.0031
Q9	56,455	4,352	6.19	5,567.59	5,351.06	0.0216
Q10	56,313	1,624	5.11	2,793.36	2,378.54	0.0211
Q11	66,194	6,022	7.04	8,505.63	8,347.99	0.0034
Q12	54,076	2,181	5.57	2,797.34	2,752.34	0.0133
Q13	63,675	6,448	7.06	9,579.11	9,254.61	0.0039
Q14	64,209	6,196	7.28	8,754.47	8,646.37	0.0023
Q15	41,550	1,975	4.82	2,833.06	2,662.82	0.0512
Q16	70,629	4,508	6.14	6,558.34	6,368.81	0.0108
Q17	69,761	5,583	6.86	7,920.16	7,813.37	0.0043
Q18	69,787	4,457	6.58	6,066.97	5,954.35	0.0060
Q19	58,847	5,250	6.98	7,503.24	7,462.99	0.0036
Q20	69,889	3,011	5.52	5,112.42	4,359.56	0.0187
Q21	50,195	6,133	7.30	8,222.99	7,800.27	0.0027
Q22	70,710	4,180	6.24	5,952.90	5,765.72	0.0138
Q23	66,060	1,797	4.07	2,412.82	2,305.62	0.0961
Q24	69,270	2,457	5.02	3,411.67	3,290.48	0.0429
Q25	67,227	1,845	4.72	2,527.01	2,446.00	0.0363
Q26	65,653	2,565	5.46	3,384.11	3,285.10	0.0168
Q27	67,248	6,078	6.86	8,670.41	8,452.96	0.0069
Q28	62,401	2,128	4.69	4,703.95	3,577.41	0.0278
Q29	58,146	3,177	5.90	4,352.74	4,218.20	0.0160
Q30	62,492	4,029	6.11	6,909.23	5,874.98	0.0126
Q31	69,045	1,108	4.09	2,111.84	1,547.16	0.0474
Q32	60,222	468	2.66	562.04	629.03	0.2432
Q33	63,027	6,927	7.42	8,920.21	8,574.11	0.0036
Q34	69,694	1,071	4.09	1,749.06	1,410.63	0.0405
Q35	66,360	495	2.91	563.81	612.00	0.1979

Abbreviation: OTU, operational taxonomic unit.
